# Rotating Circular Micro-Platform with Integrated Waveguides and Latching Arm for Reconfigurable Integrated Optics

**DOI:** 10.3390/mi8120354

**Published:** 2017-12-01

**Authors:** Jonathan Briere, Mohannad Y. Elsayed, Menouer Saidani, Martin Bérard, Philippe-Olivier Beaulieu, Hadi Rabbani-Haghighi, Frederic Nabki, Michaël Ménard

**Affiliations:** 1 Aeponyx Inc., Montreal, QC H3C 4J9, Canada; mberard@aeponyx.com; 2 Department of Computer Science, Université du Québec à Montréal, Montreal, QC H2X 3Y7, Canada; beaulieu.philippe-olivier@courrier.uqam.ca (P.-O.B.); hadi.rabbani@gmail.com (H.R.-H.); menard.michael@uqam.ca (M.M.); 3 Department of Electrical Engineering, École de Technologie Supérieure, Montreal, QC H3C 1K3, Canada; mohannad.elsayed@mail.mcgill.ca (M.Y.E.); menouer.saidani@gmail.com (M.S.); frederic.nabki@etsmtl.ca (F.N.)

**Keywords:** microelectromechanical systems (MEMS), electrostatic actuator, latch, micro-opto-electro-mechanical systems (MOEMS), micro-platform, optical switch, scanning, integrated optics, silicon-on-insulator (SOI) MEMS, optical waveguide

## Abstract

This work presents a laterally rotating micromachined platform integrated under optical waveguides to control the in-plane propagation direction of light within a die to select one of multiple outputs. The platform is designed to exhibit low constant optical losses throughout the motion range and is actuated electrostatically using an optimized circular comb drive. An angular motion of ±9.5° using 180 V is demonstrated. To minimize the optical losses between the moving and fixed parts, a gap-closing mechanism is implemented to reduce the initial air gap to submicron values. A latch structure is implemented to hold the platform in place with a resolution of 0.25° over the entire motion range. The platform was integrated with silicon nitride waveguides to create a crossbar switch and preliminary optical measurements are reported. In the bar state, the loss was measured to be 14.8 dB with the gap closed whereas in the cross state it was 12.2 dB. To the authors’ knowledge, this is the first optical switch based on a rotating microelectromechanical device with integrated silicon nitride waveguides reported to date.

## 1. Introduction

Photonic integrated circuits are receiving increasing attention as a promising technology for the next generations of high-speed, low-loss, low-power consumption, and low-cost communication and sensing systems. Concurrently, progress in microelectromechanical systems (MEMS) has helped the miniaturization and realization of complex optical components on-chip such as lenses, mirrors, filters, beam splitters, and gratings [1]. These elements are commonly referred to as micro-opto-electro-mechanical systems (MOEMS) [1]. The emergence of silicon-on-insulator (SOI) wafer batch processing technology has accelerated the development of high performance and reliable passive components including low-loss waveguides, power splitters and multiplexers [2,3]. Demand for high-speed optical communications pushed the development of several novel optical systems, e.g., micromirror arrays for large optical space switches [4], continually tunable optical delay lines [5], and reconfigurable optical add/drop multiplexers (ROADMs) [6]. MEMS mirrors are promising candidates for applications where size, weight, power consumption, and cost are critical parameters [7,8,9,10,11,12,13,14,15]. They have been used in several applications to variably deflect an optical beam, e.g., wave front control [8], scanners [9,10] and a variety of small and large-scale optical switches [10,11,12,13].

MEMS actuators are receiving increasing attention for co-integration with optical components leading to several innovative photonic devices [16,17]. They can be used for out-of-plane actuation as in [18], or in-plane actuation as in [19,20,21,22,23,24,25,26,27,28,29]. Rotary comb actuators are one key actuator type. In [19,20,21,22], in-plane rotary comb-drives suspended by serpentine flexures were demonstrated to achieve larger rotation angles than those suspended by clamped straight beams. The serpentine flexures enabled the in-plane rotary comb-drives to mechanically rotate over 9° in each angular direction at driving voltage bias of less than 60 V. However, the devices exhibit relatively low resonance frequencies of ~410 Hz, which limits the speed of operation. In [23], the design of a real pivot formed by a double-clamped beam for the rotational tuning structures in MEMS tunable lasers was presented. The structure achieved a rotation of ~4.7° with a 75 V actuation voltage. In [24], rotary comb-drives with deflection angle up to 2.8° were demonstrated. In [25], rotary comb-drive electrostatic actuators with virtual pivotal point of rotation were designed and fabricated for applications in external cavity tunable lasers in a Littman configuration. A maximum rotation of ±1.5° at 190 V was achieved. In [26], a long-arm comb-drive rotary actuator with an externally mounted large mirror is presented. Static characterization measurements show that the actuator can achieve a rotational angle of 3° with an applied voltage of 130 V. In [27], a MEMS switch based on a rotary electrostatic comb actuator was presented. The switch uses a 50 V DC actuation voltage and exhibits a 0.774° rotation angle. In [28], rotary comb-actuators are used to realize a micro-gripper. It achieves ~2° angular displacement with 100 V actuation voltage.

Accordingly, this work presents the development of an innovative laterally moving rotational micro-platform that provides a significant lateral deflection angle using a compact footprint. The micro-platform is integrated under optical waveguides to control the direction of propagation of light within a die. Prior work on the MEMS actuator was briefly presented in [29]. This article presents a detailed analysis of the optimization as well as testing results of the fabricated structures. To introduce the different aspects related to the micro-platform, the envisioned system and micro-platform are discussed in Section 2. Then, the micro-platform designs are described in Section 3. The test setup is presented in Section 4. The experimental results of the different implemented devices as well as the limitations of each device are then reported in Section 5. This section also includes optical testing results of a crossbar optical switch implemented using the platform. Finally, the results are discussed in Section 6 along with the conclusion.

## 2. System Overview

### 2.1. Targeted Micro-Opto-Electro-Mechanical System

**Figure 1:**
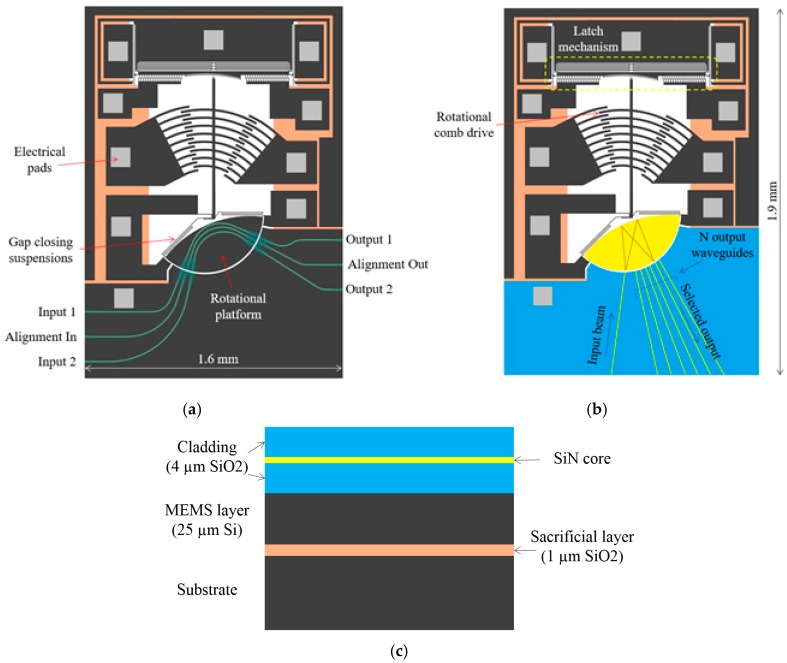
Example applications of the proposed actuator: (**a**) schematic of the crossbar switch; (**b**) schematic of the 1 × N mirror-based switch; (**c**) cross-section of the key material stack.

The micro-platform presented here was designed to be integrated with optical waveguides on the same chip using a novel fabrication technology [26,30]. Such integration is challenging because MEMS devices incorporate suspended movable parts, which means that light propagating inside a waveguide might face discontinuities resulting in significant optical losses. Careful engineering is required in this case in order to minimize losses at these interfaces and optimize the propagation of light. By taking advantage of a silicon nitride (Si_3_N_4_) core enclosed in a silicon oxide (SiO_2_) cladding, the platform allows to create single mode low propagation loss waveguides over standard silicon-on-insulator (SOI) substrates. Silicon nitride is an excellent choice to operate in the telecom band around 1550 nm. This is because it can be annealed to drive out hydrogen in order not to suffer the N-H and Si-H bonds absorption at around 1520 nm [31,32]. Silicon nitride can also be used within the datacom range down to 500 nm where silicon suffers absorption below 1100 nm. Furthermore, this waveguide structure can easily be tailored to different applications by adjusting the thickness of the different layers, such as polarization insensitive filters [33,34], and the various deposition methods available for these materials provide flexibility in the definition of the fabrication process. Figure 1 shows schematics of two example applications of the micro-platform as a 1 × N switch and a crossbar switch cell as well as a cross section illustrating the material stack.

In the 1 × N switch, the micro-platform lies under a planar optical waveguide, as shown in Figure 1b, and implements a rotational solid-immersion MEMS mirror [35], where light propagates in the nitride core layer. The input waveguide brings the light signal to the movable MEMS mirror structure by crossing the air gap between the waveguides and the planar waveguide. The light beam starts to diverge inside the planar waveguide until it reaches the curved back facet of the mirror that reflects the light toward to the output waveguide while refocusing it.

The mirror can reflect the light through total internal reflection using the effective index contrast between the air behind the mirror and the silicon nitride planar waveguide or using a reflective coating on the back facet of the planar waveguide. The ability to rotate the mirror allows for positioning the output focal point of the light, and consequently, selecting the output waveguide. The light beam crosses the air gap between the movable mirror part and the anchored waveguide once more on the output side.

For the crossbar switch cell [36], the micro-platform carries Si_3_N_4_ waveguides cladded with 4 µm SiO_2_ layers. Inverted tapers are located at the edges of the chip to improve coupling with lensed fibers by engineering the optical mode and on each side of the gap defining the rotational platform to minimize diffraction and increase alignment tolerance, thus improving on optical losses through the gap. The cladding is widened in these regions to ensure that the expanded optical mode does not interact with the cladding boundaries. The input waveguide brings the light signal to the movable MEMS structure by crossing the air gap between the waveguides. The switch has three input and output waveguides. The middle waveguide serves for alignment purposes during testing and would not be required in a switch fabric. In the bar state, when the platform is not rotated and only the gap-closing actuator is activated, the signal from input waveguides 1 and 2 are transferred to the matching outputs.

To put the switch in the cross state, the platform is rotated by 5° counter clockwise. Then, input 2 is connected to output 1 by the waveguide in the middle of the platform when the gap is closed. The other waveguides remain unconnected. Nevertheless, these two states are sufficient to build a crossbar matrix by fabricating an array of this device. The light beam crosses the air gap between the movable mirror part and the anchored waveguide once more on the output side.

An electrostatically actuated gap-closing mechanism that translates the MEMS micro-platform is utilized in order to reduce the size of the air gap after rotation and consequently limit the optical losses due to the expansion of the light beam in the air gap, which is desirable for both proposed applications. The Si_3_N_4_ core thickness and waveguide dimensions are optimized for the requirements of each particular application.

### 2.2. Micro-Platform

Since the optical properties of a material depend on its temperature, a power efficient MEMS actuation mechanism is essential in order to maintain the temperature of the optical components constant, precluding the use of thermal actuation. Magnetic and electrostatic actuation mechanisms were considered for the device. However, magnetic actuation requires components that are relatively large compared to the proposed MEMS device. On the other hand, electrostatic actuation was found to be suitable for the targeted dimensions. In addition, electrostatic actuation does not require any DC current and therefore suits the power requirements of the application. The selection of the output waveguides can then be controlled through the electrostatic actuators using a standard high voltage driver (e.g., [37]).

Besides the planar rotation that is achieved with the bi-directional comb actuator shown in Figure 1, two additional functions were implemented in the MEMS structure to improve the performance of the whole MOEMS system. The first one described previously in Section 2.1 is the ability to close the gap between the movable and the anchored part of the MEMS, and consequently reduce the optical losses associated with crossing the air gap. The second feature that was implemented is a latch that enables the capability of selecting an output channel (i.e., waveguide) and holding the rotating structure in position. This is relevant for many applications, including in telecommunications, where the MEMS needs to remain in a fixed position for long periods. This allows for the device to be used without an actuation voltage after locking it in position, consequently saving power.

Moreover, the latch system needs be protected from a potential power (i.e., actuation voltage) failure by using a latch lock that secures the latch in position in order to keep the optical communication active through the selected channel. Theoretical electrostatic equations and pull-in voltage approximation were used as starting points to create the different actuator designs of the rotational actuator, gap closer, latch, and latch lock. These designs were then optimized using finite-element methods (FEM) simulations. The device designs are outlined in the following section.

## 3. Device Designs

### 3.1. Rotational Actuators

The MEMS rotational actuators are required to achieve a wide angular motion, in order to allow for a large number of waveguides to be supported by the 1 × N switch. The structures also need to be compact and fast to achieve a small system footprint and sufficiently high switching speed. Three different actuator structures were designed and optimized for maximum displacement by using both FEM simulations and experimental validation. The FEM simulations were carried-out using the software ANSYS (version 18.0, ANSYS, Inc., Canonsburg, PA, USA).

As demonstrated by the results reported below, the position of the virtual pivot assumed in the design of the circular comb drive has a significant impact on the quality of the motion (i.e., how close it can follow a perfect circle) and on the comb drive geometry. The remaining part of this section presents a detailed description of the geometry of the different actuators investigated for this platform. The experimental measurements are discussed in Section 5.

Every design includes a micro-platform with a radius of 300 µm covering an arc of 135°. All the prototypes presented here feature a 3 µm wide and 70 µm long anchor that also serves as a flexural spring. These anchor dimensions were selected to achieve a good trade-off between the quality of the rotational motion and the actuation voltage. In addition, the initial platform has been built with a 45° facet on one side to facilitate subsequent optical tests using the MEMS structural layer to guide light. Moreover, strategically placed dimples were added on each design to act as stoppers for the rotation of the platform, thus preventing the actuator with the movable fingers to collapse.

**Figure 2:**
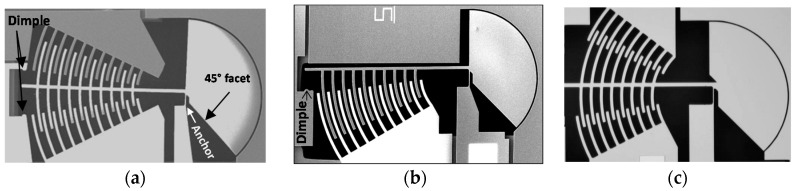
Micrographs of: (**a**) the initial design, (**b**) the 2nd version one-sided optimized actuator design, and (**c**) the 3rd version two-sided actuator design.

The first design implemented is shown in Figure 2a. The circular comb drive of this design was created by assuming that the pivot point is in the middle of the anchor. Each circular finger has a width of 10 µm in order to have sufficient structural strength. The asymmetry in the rotation of the circular comb, which is a consequence of the bending of the anchor, was compensated by creating asymmetric spacing on both sides of the fingers to make sure that the actuation is balanced and to prevent unintentional early electrostatic pull-in. The interior gap (i.e., the one closest to the mirror) between the moving and fixed fingers on the left actuator is 9 µm whereas the outer gap (i.e., the one closest to the latch) is 13 µm. On the right side, the interior and outer gaps are 10 µm and 12 µm, respectively. This makes the pitch between two consecutive fingers on either of the moving comb or the fixed comb to be 42 µm. The total length of the mast is 650 µm and the longest finger external edge is positioned at 583 µm, both measured from the edge of the anchor.

The second design is shown in Figure 2b (the one sided circular comb). In this version, the rotation pivotal point was positioned at 2/3 of the length of the anchor from the anchored edge based on simulations, as opposed to the middle of the anchor in the first design. The final position of the rotation point was obtained through an iterative analysis of the simulation results, and the result is in good agreement with similar works presented in the literature [23]. The center point used to create the circular comb drive was then moved to the new pivotal point. The distance between the edge of the anchor and the external edge of the longest finger on this design is 655 µm. The width of the fingers was increased to 12 µm in order to increase their strength and reduce their flexural bending. The pitch between fingers is 46 µm in this design.

The third design, illustrated in Figure 2c, uses the same estimated rotation point (2/3 of the length of the anchor) that allows the device to rotate almost perfectly. The right side actuator was added in order to double the rotation angle. The main mast holding the actuator fingers is 700 µm long and 12 µm wide to prevent bending. The longest fingers are positioned at 600 µm from the anchor and the remaining fingers are equally spaced by 29 µm. The width of each finger is 12 µm as in the second design. The distance between the rotational and fixed fingers was optimized to achieve the largest angular displacement possible by preventing any premature contact between the moving and static fingers. These distances are more aggressive compared to the two previous design and vary from 7 µm to 10 µm. A stopper was implemented at the top of the mast using 3 µm radius dimples to prevent stiction of the structure. The actuators are designed to allow a free rotation of over 10° in each direction without pull-in.

**Figure 3:**
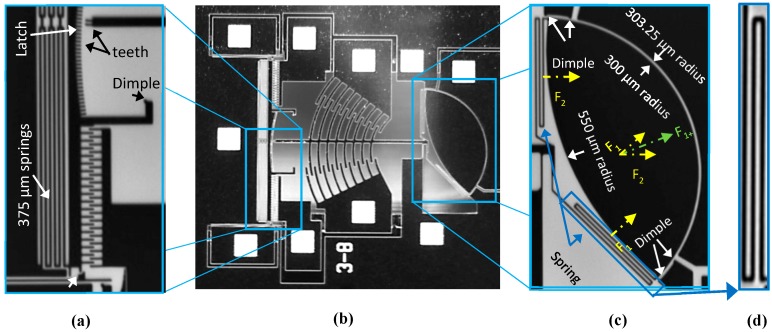
Scanning electron microscope (SEM) micrographs of the third design: (**a**) latch and latch lock, (**b**) whole device illustrating the curved back facet, (**c**) gap-closing structure where the yellow-dashed arrows show springs displacements, and the green-dashed arrow shows the gap closer resulting displacement, and (**d**) spring.

Note that the back facet of the platform was modified as shown in Figure 3 such that the outer interface of the mirror to be added above the micro-platform focuses the reflected light beam in the output waveguide in order to suit the 1 × N switch application.

Three additional structures were added to the third design iteration: a latch, a latch lock, and a gap closer. Figure 3b shows a scanning electron microscope (SEM) micrograph of the whole device including the added structures. Zoomed images of the latch and latch lock are shown in Figure 3a, whereas the gap closer is shown in Figure 3c with a zoomed view of its springs in Figure 3d. The latch and latch lock structures allow the micro-platform to lock at a certain position without requiring a voltage bias. These structures are described in the following subsections.

### 3.2. Latch and Latch Lock

The latch is positioned at the end of the actuator mast. As shown on Figure 3b, its locking mechanism consists of a set of teeth that follows the curve described by the mast of the circular comb drive during the rotation of the micro-platform. Through electrostatic actuation, the latch translates towards the mast such that the two teeth at the end of the mast lock inside the closest teeth of the latch. The alternative would have been to design a latch that is initially in a locked position. However, this design would have required a 15 µm cavity at the initial position of the structure to ensure that it would be released during fabrication. This calculation is done knowing that the minimum dimension that can be etched in the fabrication process used to implement the prototypes is 3 µm and that etch areas must be defined at three positions around the pair of teeth defined at the end of the mast: one on both sides of the teeth and one between them. Moreover, the minimum feature size of the teeth is also 3 µm, making a total of five times 3 µm, and hence the total 15 µm width of the whole structure. The length of the mast from the anchor to the latch teeth was set to 700 µm to leave additional space for the latch actuator beyond the circular comb drive. The latch teeth are 3 µm wide and 11 µm long. To improve precision, they are designed to follow a 714 µm radius at their base and a 703 µm radius at their tip. All of them are centered at the micro-platform rotation point so that the mast teeth are always parallel to the latch teeth in order to lock properly. The latch is anchored through symmetrical support beams acting as springs to enable the directional movement of the latch. Each beam is 375 µm long and 3.5 µm wide with 3 µm spacing between them. This configuration allows an angular latching precision of 0.25° over the entire motion of the platform. The latch actuator is a directional comb drive made of 38 straight teeth of 20 µm in length and spaced by 3 µm. The available displacement space in front of the fingers inside the actuator is 12 µm. The translational movement of the latch is limited by a stopper to 11 µm. This leaves a 1 µm spacing at the maximum displacement such that even if the latch goes into pull-in, it will not touch the actuator and result in a short-circuit. The latch actuators are symmetric and positioned on both sides of the mast to ensure that the motion is stable and effective. An additional latch lock structure was added to hold and lock the latch into position with no applied voltage. It is positioned on each side of the latch and makes a translational motion orthogonal to the latch. It includes an 11 µm movable pin located at the end of a 300 µm long and 3 µm wide beam, as shown in Figure 3b. This beam is actuated to allow the displacement of the latch and it is then returned to its initial position to lock the latch when the latch actuation voltage is removed. A dimple was added on the side of each actuator of the latch lock to prevent stiction at pull-in. These two structures operate simultaneously to release and lock the latch.

### 3.3. Gap Closer

The gap closer enables a translational displacement of the platform towards the fixed part of the substrate in order to reduce the air gap between the waveguides on each side and consequently minimize the optical losses. Two sets of suspension springs link the ends of the micro-platform to the main mast as shown on Figure 3c. The positions of their anchors are designed to prevent any discontinuity along the back-facet of the platform that could cause optical losses for the 1 × N mirror-based switch application. In addition, their positions allow reliable translational motion of the platform. It is worth mentioning that the gap-closing movement towards the fixed part of the waveguides maintains the rotation angle. Each of the suspension springs is composed of three beams that are 205 µm long and 3 µm wide. This ensures minimal lateral displacement, which is necessary for the platform to sit on the dimples after closing the air gap. Two 3 µm dimples acting as stoppers were added on both sides of the actuator to prevent stiction or electrical contact of the platform with the static part of the gap-closing actuator, while minimizing the gap. The edges of these two dimples are 3 µm away from the external edge of the micro-platform and placed at a certain distance from the fixed part containing the waveguides allowing final gaps as low as 250 nm after activating the actuator.

The back facet of the platform was engineered to compensate the divergence of the beam inside the planar waveguide for the 1 × N mirror-based switch application. For this purpose, the 45° facet was replaced by a circular back facet with a 550 µm radius facing the front facet (as shown in Figure 3c), such that the incoming light beam reflected by the curved back facet is focused back into the exit waveguides above the platform. The optical losses due to the divergence of the beam are thus minimized. The radius of the front facet of the mirror remained 300 µm as the other designs.

## 4. Test Setups

**Figure 4:**
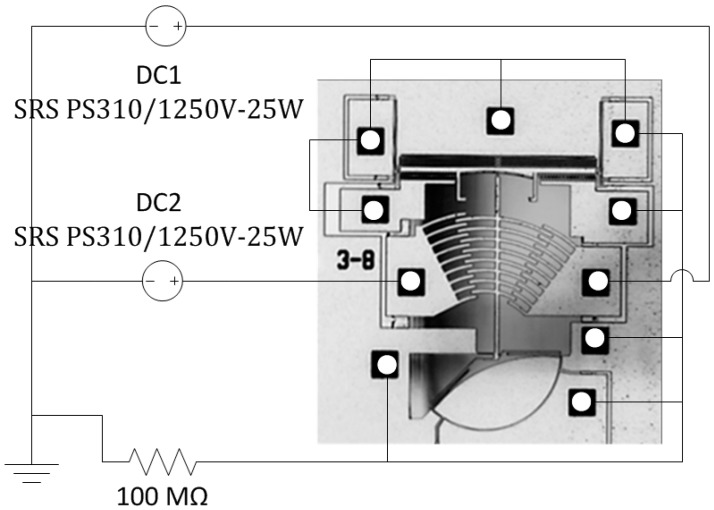
Test setup for the rotational actuation of the platform.

The test setup to characterize the rotational motion of the platform is shown in Figure 4. The fabricated MEMS platforms were tested using a probe station with four DC probes and a high-resolution camera (Basler Ace2000-340kc, Basler AG, Ahrensburg, Germany) to capture images at the different actuation voltages. One rotation actuator is connected to a high voltage power source (SRS PS310/1250V-25W, Stanford Research Systems, Sunnyvale, CA, USA) with a large resistance of 100 MΩ in series to prevent high current if the actuator touches the structure accidently during displacement. The unused actuators are all grounded using probes or wire bonding to ensure that they do not accumulate charges, which may result in unintentional electrostatic forces leading to undesired displacement of the movable structures. The probes are positioned on the metal pads and a set of pictures is then taken, one for each value of the actuation voltage. The pictures are then post processed using image processing software, in order to calculate the angular motion for the different actuation voltages. Rotation in the other direction was tested similarly by connecting the voltage source to the opposite actuator. The other actuators (i.e., latch, latch lock, and gap closer) were tested in a similar fashion.

**Figure 5:**
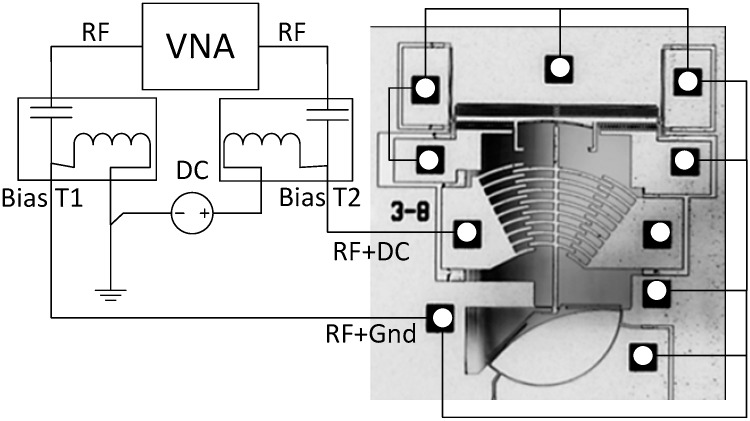
Test setup for the resonant frequency measurement of the platform.

The test setup to characterize the resonant frequency of the platform is shown in Figure 5. The resonant frequency tests are done using a vector network analyzer (VNA) (Agilent E5061B, Agilent Technologies, Santa Clara, CA, USA) and a custom printed circuit board (PCB). Bias tees are used to bias the structure and to overlay the VNA signals. The device die is wire-bonded in order to connect the biasing and AC signals required. The resonance measurement is performed in a 1 mTorr vacuum environment using a custom made vacuum chamber in order to improve the quality factor of the resonance, since the quality factor of the device is too low to allow for such a measurement to be done in air due to excessive air damping.

For optical measurements, the fabricated crossbar switch was mounted on a PCB and wire bonded. The board was placed between two 3-axis micro-positioning stages holding tapered lensed fibers and it was connected to two high voltage sources to control the MEMS. Light from a laser operating at 1550 nm was coupled to the waveguides on the chip and the output power was measured with a power meter. The input polarization was optimized with a polarization controller.

## 5. Results

Prototype MEMS platforms were fabricated in a commercial SOI process (SOIMUMPs from MEMSCAP, Crolles, France) [38]. This process allows the fabrication of the MEMS itself, but it does not include the optical stack above the MEMS platform. It nonetheless allows to design the MEMS platform, and to characterize its motional performance and features for the MOEMS system described in Section 2.1.

The experimental results for the three designs described above are presented in this section. Observations on the fabrication process are given in Section 5.1. The following subsections present the measured responses of the different fabricated structures. Note that more measurement details are presented for the third design as it represents the optimized design for the targeted system.

### 5.1. Fabrication Process Characterization

**Figure 6:**
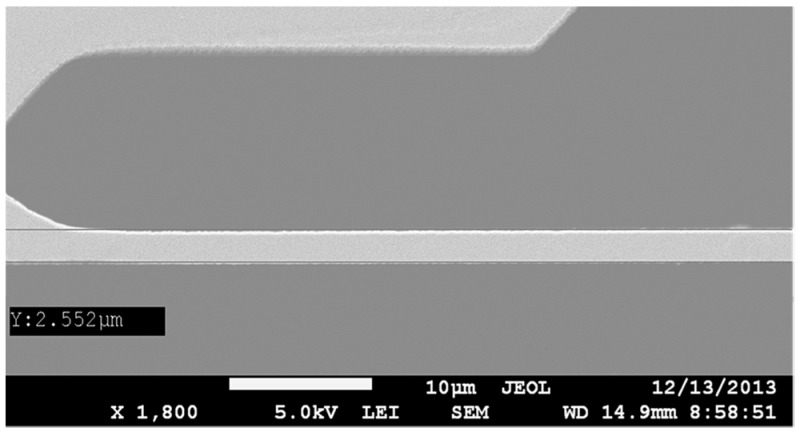
SEM micrograph showing the measured anchor width.

The main rotation anchor was first laid out with three different width values of 3 µm (SOIMUMPs technology limitation), 3.5 µm and 4 µm, and a single length of 80 µm. In this fashion, the critical parameters of the anchoring structures can be determined, which is key to the rotational motion of the platform. A SEM micrograph shows that the resulting fabricated anchors have widths of 2.5 µm, as shown in Figure 6, 3.2 µm and 3.9 µm, respectively. The anchor of 3.2 µm proved to be sufficiently strong to provide a high fabrication yield, while allowing for a wider rotation for a given voltage range in comparison to the 3.9 µm anchor. Accordingly, it was selected as the reference anchor width to use for all designs.

Knowing that the Young modulus, the Poisson’s ratio and the shear modulus of silicon change significantly with the crystal orientation [39,40], investigations of the impact of these properties on the actuation voltage were done on the final design.

### 5.2. First Design

**Figure 7:**
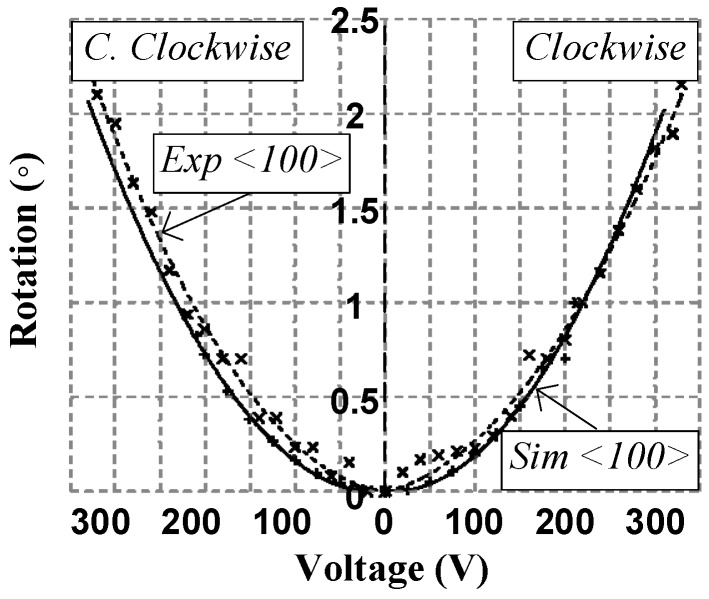
Measured and simulated platform angular displacement for left and right actuation of the first design where the “+” data points represent simulated data, the solid line is a fit to the simulated data, the “×” data points represent experimental measurements, and the dotted line is a fit to the experimental measurements.

Using a test setup similar to the one shown in Figure 4, the first design achieved 2.1° of rotation for clockwise (CW) actuation at 330 V and 2.25° of rotation for counter clockwise (CCW) actuation at 330 V, as shown in Figure 7. It is important to indicate that the fabrication of this design was carried out in the <100> silicon crystalline plane since the orientation does have an impact over the Young’s modulus, the shear constant and the Poisson’s ratio. The non-optimized rotation point of the comb drive caused the fixed and mobile fingers to touch, which limited the rotation angle. As explained above, the rotation point was corrected to prevent this problem in the second and third designs.

### 5.3. Second Design

**Figure 8:**
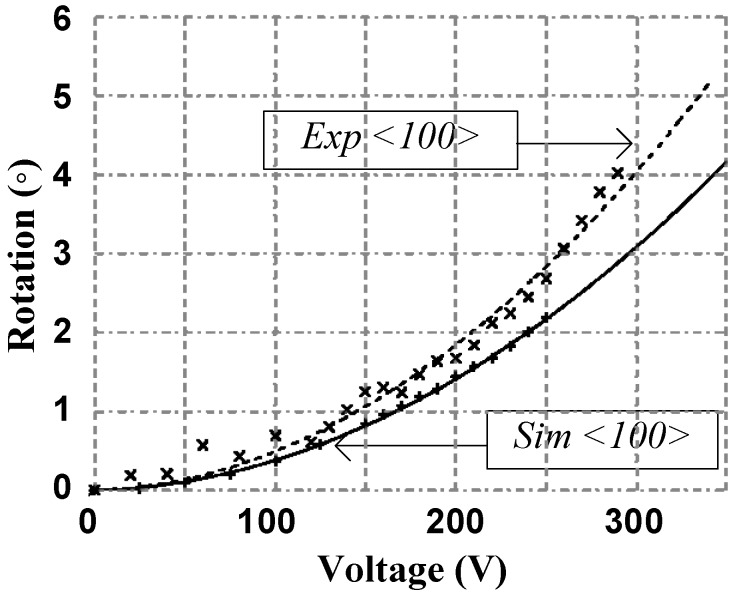
Measured and simulated platform angular displacement of the second design with fitting curves where the “+” data points represent simulated data, the solid line is a fit to the simulated data, the “×” data points represent experimental measurements, and the dotted line is a fit to the experimental measurements.

The second design implemented the improved circular comb actuator design with the optimized rotation pivotal point. The actuator rotation was characterized using the test setup shown in Figure 4. Figure 8 shows the measured rotation angle of the actuator. This design exhibits a 4° maximum rotation at 290 V for a fabrication following using the <100> silicon crystalline plan. The actuation voltage is lower than that of design 1 due to the difference in the dimensions of the comb fingers.

### 5.4. Third Design

**Figure 9:**
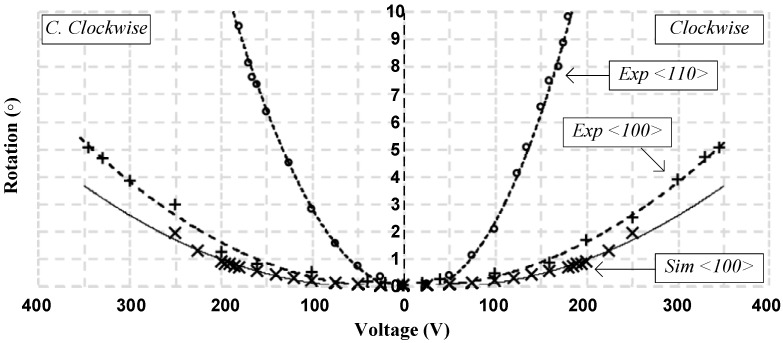
Platform angular displacement for left and right actuation of the third design with fitting curves showing more than ±5° both sides for <100> anchor orientation and ±9.5° for <110> anchor orientation where the “×” data points represent simulated data, the solid line is a fit to the simulated data, the “+” data points represent experimental measurements on the <100> fabrication plane, the “o” data points represent experimental measurements on the <110> fabrication plane, and the dotted lines are a fit to the experimental measurements.

The circular comb drive design of the third design proved to yield the best results. The angular displacement reached 5° for each side with a 350 V actuation voltage when the anchor is defined parallel to the <100> crystal plane of the silicon structural layer, which is the same orientation as the other designs. This resulted in a total angular motion of more than 10° when both actuators are used, as shown in Figure 9. Moreover, the maximum angular displacement almost doubles to reach 9.5° for each side and the actuation voltage required to reach this maximum is 180 V when the anchor is designed along the <110> crystal plane (rotated 45° compared to the other designs). As a result, it becomes possible to cover a total of more than 19° as illustrated in Figure 9. This is due to the anisotropic nature of the Young’s modulus in single-crystal silicon [39,40].

**Figure 10:**
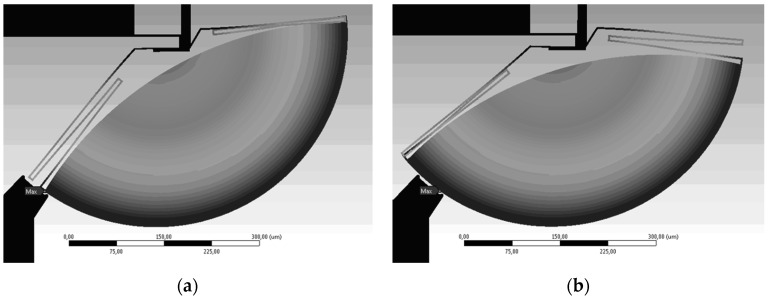
Simulated resonance of the structure: (**a**) First extremum, and (**b**) second extremum.

**Figure 11:**
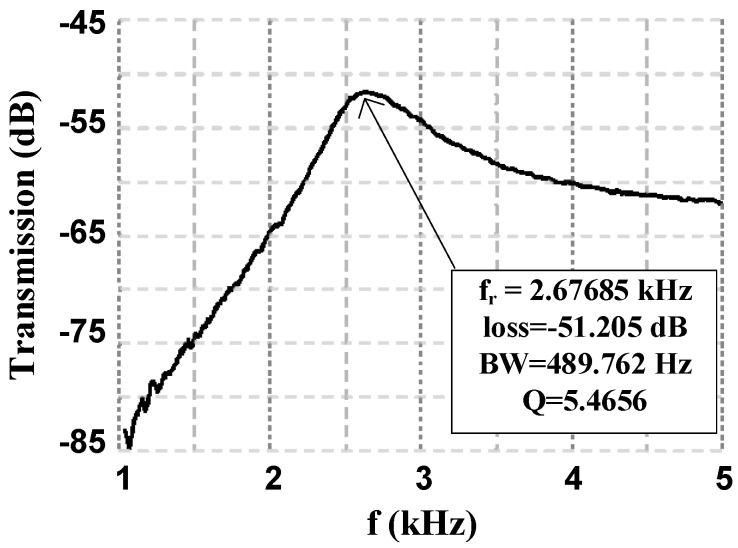
Measured resonance characteristics of the third design at 1 mTorr vacuum level.

The resonant characteristics of the platform were obtained using the test setup shown in Figure 5. The fundamental resonant mode is that of the gap closer structure. The two extrema of the resonance mode shape are illustrated in Figure 10. The device exhibits a resonant frequency of 2.68 kHz with a quality factor of 5.47 in vacuum as shown from the measured transmission curve in Figure 11. This value is directly linked to the gap closer such that a modification to the gap closer will impact the resonant frequency of the MEMS. A modification made to the anchors of the gap closer is under investigation to increase the resonant frequency and set it such that the first resonant frequency to appear is the one coming from the rotation of the platform, which is close to 3.5 kHz.

### 5.5. Latch and Gap Closer

**Figure 12:**
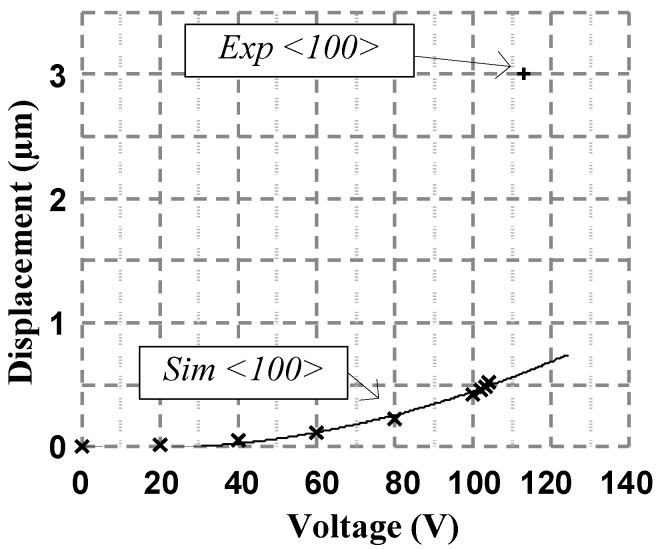
Simulation and experimental gap closer displacement vs the actuation voltage for <100> anchor orientation where the “×” data points represent simulated data, the solid line is a fit to the simulated data, and the “+” data points represent experimental measurements.

The experimental results of the gap closer yielded that the structure can minimize the air gap and close the 3.25 µm initial separation with an actuation voltage of 113 V. The measured pull-in voltage is a bit lower than the FEM simulation results, as shown in Figure 12. The simulated resonant frequency of the gap closer is of 4.30 kHz. The experimental resonant frequency is 2.68 kHz as stated before. The difference between the simulation and measured results of the pull-in voltage and resonance frequency can be attributed to the fact that the width of the fabricated anchors of the gap closer are thinner than the designed width which results in lower spring constants. No stiction of the platform was observed during measurements, and it was free to open and close rapidly and repeatedly during the experimental tests.

**Figure 13:**
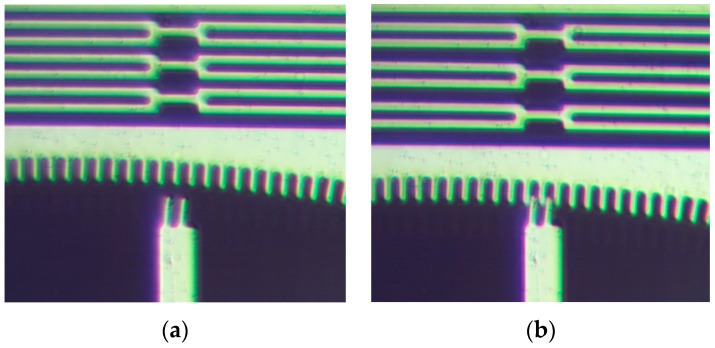
Locking mechanism: (**a**) Initial position and (**b**) latched position.

**Figure 14:**
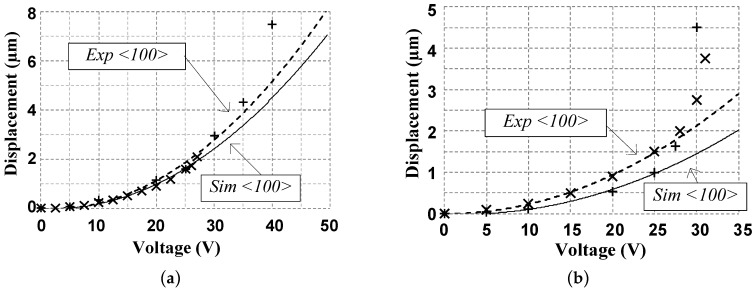
Latch actuation for <100> anchor orientation showing (**a**) locked latch at 40 V and (**b**) open lock at 30 V where the solid line is a fit to the simulated data, and the dotted line is a fit to the experimental data.

Moreover, the experimental results of the latch and latch lock validated the operation of the structures. Figure 13 shows the initial and latched positions of the latch teeth. The displacement needed to firmly lock the teeth of the latch is about 7 µm and the displacement needed to pull the latch lock away from the latch is 3 µm. The resulting measured actuation voltage for the latch is 40 V and the actuation voltage for the latch lock is 30 V. Both of these voltages are in good agreement with the simulations as demonstrated in Figure 14a,b. Simulation results yield a resonant frequency of 2.99 kHz for the latch structure.

### 5.6. Optical Measurements of a Crossbar Switch Cell

As an example application, measurement results from a crossbar switch cell implemented using the optimized rotating platform design are presented to demonstrate the viability of the proposed system. The switch was fabricated in a custom microfabrication technology involving a mixture of surface and bulk micromachining to integrate the waveguides and the MEMS structures.

**Figure 15:**
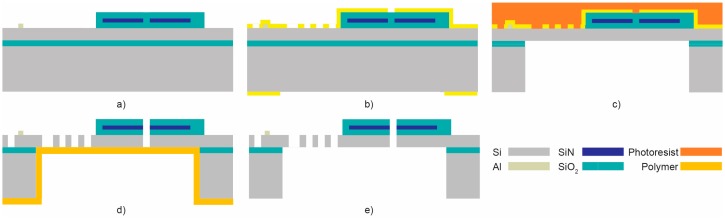
Simplified process flow (**a**) silicon-on-insulator (SOI) with patterned waveguides and Al pads, (**b**) front and back patterned Cr mask, (**c**) front protective layer and backside etch, (**d**) backside polymer deposition and micro-opto-electro-mechanical systems (MOEMS) etch, (**e**) MOEMS release by stripping polymer [33].

A simplified fabrication process flow of this cross bar switch cell is illustrated in Figure 15 [36]. The process starts with an SOI wafer with a 25 µm silicon device layer. First, a 4 µm layer of silicon dioxide and a 200 nm layer of silicon nitride are deposited through plasma-enhanced chemical vapor deposition (PECVD) forming the bottom cladding and core layer of the waveguides, respectively. Then, the silicon nitride is patterned using e-beam lithography to define the waveguides. Afterwards, a 4 µm layer of PECVD silicon dioxide is deposited and patterned using a chromium (Cr) hard mask and reactive ion etching (RIE) going through the both the bottom and top claddings. A 300 nm layer of aluminum is then sputtered and wet patterned to form the pads for electrical contacts. These steps result in the structure shown in Figure 15a. Chromium hard masks for patterning the top structures and bottom release trenches are then patterned using dry etching as shown in Figure 15b. Release trenches are then deep RIE etched through the Cr mask on the bottom side. Then, the Cr mask is dry stripped and a protective photoresist layer is applied to the top side. The buried oxide layer is then wet etched in 49% hydrofluoric acid while the top is protected by the photoresist layer, as shown in Figure 15c. Afterwards, the photoresist layer is stripped and a layer of polymer is deposited from the backside to hold the structures in place after etching. Then, the structures are dry etched from the top side using the Cr mask to go through the waveguide layers and structural silicon in order to define the optical facets and MEMS structures, as illustrated in Figure 15d. The structures are finally released in oxygen plasma to remove the polymer, as shown in Figure 15e.

**Figure 16:**
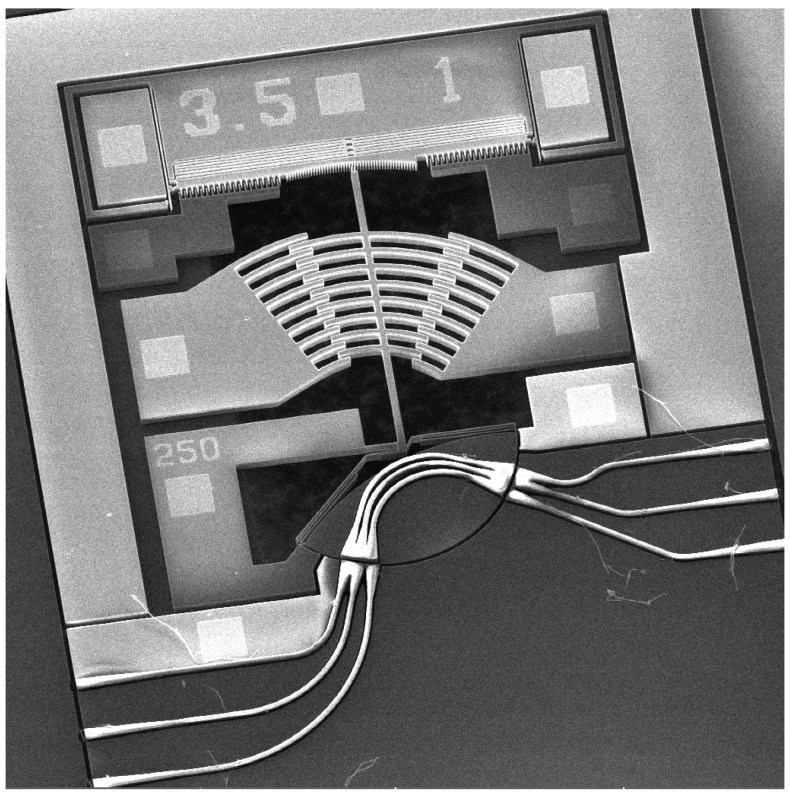
SEM micrograph of a fabricated crossbar switch [33].

Moreover, the waveguides are made of a 200 nm-thick and 1.5 μm-wide silicon nitride core, ensuring single TE mode operation. Inverted tapers where the core narrows down to 494 nm are located at the edges of the chip whereas on each side of the gap the tapers are 406 nm wide to improve coupling with lensed fibers and minimize loss due to beam expansion, respectively. The mode overlap analysis was done using optical modal overlap simulation and the inverted tapers length optimization was obtained using bidirectional EigenMode Expansion analysis; both optimizations were carried out in Lumerical MODE software (version 2017b, Lumerical Inc., Vancouver, BC, Canada). Figure 16 shows a SEM micrograph of the fabricated switch [33].

For the optical measurements, light was injected into the chip from a laser operating at a wavelength of 1550 nm. The laser was connected to a polarization controller and a lensed tapered fiber with an output beam diameter of 2.5 µm. The output signal was collected with a similar lensed tapered fiber and measured with a power meter. The input polarization state was optimized with the polarization controller to maximize the output signal. For the current design, this should correspond to the fundamental TE mode. Both lensed tapered fiber were mounted onto 3-axis micro-positioning stages to align them to the device under test.

In the bar state, both paths showed a total insertion loss of 14.8 dB once the gap was closed (to a 500 nm final gap in this device version). This value includes coupling losses in and out of the chip, propagation losses along 2 mm, and the losses in the remaining 500 nm wide gaps on each side of the platform. Further development of the fabrication process is ongoing to improve fiber coupling and the quality of the waveguides. Closing the gap in order for the platform lands on the dimples required 105 V.

Switching the platform in the cross state required the application of 118 V on the comb drive. With the gap closed, the total insertion loss was 12.2 dB. With the gap open the loss was 24.5 dB, thus the gap-closing mechanism improves the performance by more than 12 dB. This excess loss is due to the greater separation between the waveguides as well as a misalignment between the moving and fixed part of the switch when the gap is open. The crosstalk was below −40 dB in both states. Propagation losses of 0.8 dB/cm on similar waveguide configuration were calculated experimentally in test structures available from previous fabrication runs, which contribute for at least 0.2 dB loss in the actual measurements. Theoretically, the optical loss over each 500 nm air gap was simulated to be of about 1.7 dB. In addition, the typical fiber coupling to the chip in the test setup contributes a minimum of 3 dB per fiber to the insertion loss. The minimal loss budget calculated for the device for two gaps, two fiber coupling and the propagation loss of the waveguides is then 9.6 dB, which is in-line with the measured loss, especially noting that alignment losses could be higher than the minimum. These loss estimates could not be confirmed experimentally because no characterization waveguides were fabricated onto the chip.

It is worth mentioning that the proposed platform is also suitable for other optical applications, for example the 1 × N switch proposed in Figure 1b [29,30]. Notably, the waveguides patterns can be designed and optimized independently of the MEMS platform design to use single mode operation or polarization independent waveguides.

## 6. Discussion

Three different micro-platform designs were implemented and tested, each with particular characteristics. The first design demonstrated a little more than 4° of angular motion using actuators on both sides and also showed that the motion inside the comb drive was not optimized and early pull-in was observed between the fixed and movable fingers. The second design improved the comb and gave more stable control of the micro-platform over an angular coverage of 4° in one direction. The third design implementing the bidirectional optimized circular comb actuator and fabricated with an anchor along the <100> crystal orientation (same as the previous designs) demonstrated ±5° of angular displacement. Moreover, the same design fabricated with the anchor along the <110> crystal plane demonstrated significant advantages over the other two designs with respect to anchor flexibility and displacement. An angular displacement of ±9.5° was measured with pull-in-free operation. This design thus enables precise control of the platform over its entire circular displacement. Table 1 compares the designs presented here to other state-of-the-art rotary actuators. The design presented here achieves the highest rotation angle to date. The third design also includes additional features: the latch, the latch lock, the gap closer, and a circular back facet. These features are useful for realizing the targeted optical systems presented in Section 2.1. The design is realized in a relatively compact footprint of 1.3 mm by 1 mm.

## 7. Conclusions

This work presented different MEMS rotational micro-platform designs aimed at integration with optical waveguides in order to implement a MOEMS system that enables the control of the propagation direction of a light beam in waveguides.

The presented micro-platform is controlled by a circular rotational electrostatic actuator. Beyond rotation, the platform features an air gap-closing mechanism that contributes to provide low and constant optical losses at any angular position of the platform. It also features a latch mechanism to secure the position of the platform without requiring a DC biasing voltage when the appropriate rotation is achieved. The latch structure allows holding the platform in position with a resolution of 0.25° over the entire angular range of motion. The gap-closing structure reduces the initial 3 µm air gap to a final gap as low as 250 nm using a DC actuation voltage of 113 V. Dimples are utilized in the design as stoppers for the different structures in order to prevent stiction of the actuators, thus enabling reliable control. The device demonstrated experimentally free or latched in plane bidirectional angular motion of up to 19° using 180 V electrostatic actuation. The experimental resonant frequency of the main device is 2.68 kHz, which allows an operation speed on the order of ~300 µs.

The platform was integrated with silicon nitride waveguides using a custom microfabrication technology to create a crossbar optical switch as an example application and preliminary measurement results were reported. The switch operates at a DC voltage of 118 V, which is required to achieve the necessary 5° rotation. In the bar state, the switch exhibits a total insertion loss of 14.8 dB with the gap closed to 500 nm through the electrostatic gap-closing mechanism. In the cross state, the switch exhibits a total insertion loss of 24.5 dB with open gap, reducing to 12.2 dB when the gap is closed. Thus, the gap-closing mechanism improves the performance by more than 12 dB. The crosstalk was measured to be below −40 dB in both states. The performance of the device will be improved by reducing the final gap of the platform to 250 nm and then 100 nm, which will theoretically lower the MEMS air gap insertion loss to about 0.1 dB. To the authors’ knowledge, this is the first optical switch based on a rotating MEMS device with integrated silicon nitride waveguides reported to date.

## Figures and Tables

**Table 1:** Comparison of MEMS Rotational Electrostatic Actuators.

Reference	Rotation Angle (°)	Actuation Voltage (V)	Area (mm^2^)	Resonance Frequency (Hz)	Notes
[19]	±9	60	1 × 1	410	Serpentine flexures used to increase the rotation angle at the expense of reduced operating speed.
[23]	4.7	75	1.5 × 0.6	N/A	A real pivot formed by a double-clamped beam was utilized for the rotational tuning structures in MEMS tunable lasers.
[24]	2.8	100	~2 × 0.5	N/A	A movable arm 2 mm long forms the main rotating structure and can be reliably operated in the kilohertz range.
[25]	±1.5	190	~2 × 1	~1 k	Actuators with movable arms of 1.2–1.5 mm with virtual pivotal point of rotation for external cavity tunable lasers.
[26]	3	130	2 × 1	246	Long-arm (>5 mm) comb-drive rotary actuator with an externally mounted large mirror for optical applications.
[27]	~±0.8	50	~0.6 × 0.6	~8–11.1 k	MEMS switch based on a rotary electrostatic comb actuator.
[28]	2	100	~2.5 × 2	10.268 k	Microgripper based on a rotary comb actuator.
**This work**	±9.5	180	1.3 × 1	2.68 k	Circular comb actuator with latch lock and gap-closing mechanisms for reconfigurable and low-loss in-plane integrated optics.
